# Comparison of corticocancellous bone graft from the anterolateral metaphysis of the distal radius versus iliac crest for the treatment of unstable scaphoid nonunion with humpback deformity

**DOI:** 10.1186/s12891-023-07134-x

**Published:** 2024-01-02

**Authors:** Sung-Chul Shin, Nah-Yon Kim, Ho-Jung Kang, Shin-Woo Lee, Ji-Sup Kim

**Affiliations:** 1 Department of Orthopaedic Surgery, Catholic-Kwandong University, Incheon, South Korea; 2 Department of Orthopaedic Surgery, College of Medicine, Ewha Womans University Mokdong Hospital, Seoul, Republic of Korea; 3 Joint Reconstruction Center, Department of Orthopaedic Surgery, Yonsei Sarang Hospital, Seoul, Republic of Korea; 4 Department of Orthopaedic Surgery, College of Medicine, Ewha Womans University Seoul Hospital, Seoul, Republic of Korea; 5 Yonsei University College of Medicine, Seoul, Republic of Korea

**Keywords:** Nonunion, Scaphoid, Graft, Corticocancellous

## Abstract

**Background:**

Corticocancellous bone grafting from the iliac crest is acceptable treatment for unstable scaphoid nonunion with a viable proximal pole. However, harvesting graft from the iliac crest is associated with donor site morbidity and the requirement of general anesthesia. Thus, bone grafting from the anterolateral metaphysis of the distal radius (DR) can be a treatment option. However, no study has compared the clinical effect between the two grafting techniques.

**Methods:**

From 2014 to 2019, patients with unstable scaphoid nonunion with humpback deformity underwent corticocancellous bone grafting from the anterolateral metaphysis of the DR (group DR) or iliac crest (group IC). Humpback deformity was determined by evaluating the scapholunate angle (SLA) ≥ 60°, intrascaphoid angle (ISA) ≥ 45°, and radiolunate angle (RLA) ≥ 15° from preoperative radiographs and computed tomography scans. The SLA, ISA, and RLA served to gauge carpal alignment. The operative time, grip strength, active range of motion (ROM), the Modified Mayo Wrist score (MMWS), and Disabilities of Arm, Shoulder, and Hand (DASH) score were assessed postoperatively.

**Results:**

Thirty-eight patients qualified for the study (group DR, 15; group IC, 23). Union rates did not differ by patient subset (group DR, 100%; group IC, 95.7%; *P* = .827), and grip strength, ROM, MWS, and DASH score were similar between groups at the last follow-up. The operative time (minutes) was significantly shorter in group DR (median, 98; quartiles, 80, 114) than in group IC (median, 125; quartiles, 105, 150, *P* < .001). The ISA, RLA, and SLA improved postoperatively in both groups (*P* < 0.001). The degree of restoring carpal alignment, as evaluated by SLA, showed superior correction capability in group DR (median, 25.3% quartiles, 21.1, 35.3, *P* < 0.05). Donor site complications were not significantly different between the groups.

**Conclusions:**

Corticocancellous bone graft from the anterolateral metaphysis of the DR for unstable scaphoid nonunion is associated with a shorter operation time and comparable results with that from the iliac crest in regard to union, restoration of carpal alignment, and wrist function.

**Level of Evidence:**

Level III.

## Introduction

Corticocancellous bone grafting is a generally acceptable treatment for scaphoid nonunion with a viable proximal pole [1–3]. As a source of corticocancellous bone grafts for scaphoid nonunions, the iliac crest (IC) is the most common source for scaphoids with high mechanical strength [1, 4, 5]. However, harvesting IC grafts is associated with significant donor site morbidity [6–9], and it requires a second surgical field and general anesthesia.

The distal radius (DR) can be one of the sources of bone graft for treating scaphoid nonunion. Corticocancellous bone grafting from the anterolateral distal corner of the DR was introduced recently [10].

Good union rates were achieved with use of IC and DR graft in various series, and they do not seem to be influenced by the graft source [11–13]. However, previous comparative studies have focused on the union rate, and no study has compared the radiologic outcome and clinical effect between bone grafting of the IC and DR for treating unstable scaphoid nonunion. Therefore, this study aimed to compare union rates, mean time to union, radiologic and clinical outcomes, and donor site complications after corticocancellous bone grafting of the anterolateral metaphysis of the DR vs. IC for unstable scaphoid nonunion with humpback deformity. We hypothesized that union rates, mean time to union, scaphoid anatomic restoration, and wrist function are comparable between corticocancellous bone graft from the anterolateral metaphysis of the DR and IC.

## Materials and methods

### Study population and data collection

Our institutional review board approved this study and waived the need for informed consent. Between March 2014 and December 2019, patients with nonunion of the scaphoid who underwent surgical treatments were enrolled in this retrospective study. Nonunion was defined as a persistent fracture gap at least 6 months after the trauma, with bone resorption and sclerotic and/or cystic changes at the fracture site on a simple radiograph and computed tomography (CT) scan. Inclusion criteria were (1) unstable nonunion with collapse and dorsal intercalated segment instability deformity or a mean scapholunate angle (SLA) ≥ 60°, intrascaphoid angle (ISA) ≥ 45°, radiolunate angle (RLA) ≥ 15° (or divergence from the uninjured wrist by ≥ 10°); (2) open debridement and reduction of unstable scaphoid nonunion with corticocancellous bone grafting from the IC or DR; and (3) internal fixation with screws. Exclusion criteria were (1) age < 18 years, (2) stable nonunion without bone loss or displacement > 2 mm, (3) previous surgery around the ipsilateral wrist, (4) revision bone grafting owing to failure of bone healing after initial fixation, (5) avascular necrosis (AVN) of the proximal pole, (6) arthritic change on radiographic imaging (scaphoid nonunion advanced collapse stage [SNAC] ≥ I), and (7) < 2 years of follow-up.

AVN of the proximal pole was determined based on the preoperative MRI findings and the final confirmation was made at the time of surgery through direct visual inspection. All patient data were retrospectively retrieved from medical records and radiographic archives. Patient demographic information, including age at the time of injury, sex, interval of injury to surgery [14], history of smoking, pretreatment methods was collected. Patients grouped by the bone graft technique used (group DR, corticocancellous bone graft from the anterolateral metaphysis of the DR; group IC, corticocancellous bone graft from the IC).). Corticocancellous bone graft from the IC were performed from 2014 to 2017, whereas the bone graft from DR was performed from 2017 to 2019. All surgeries were performed by one senior author.

### Surgical techniques and postoperative rehabilitation

**Fig. 1:**
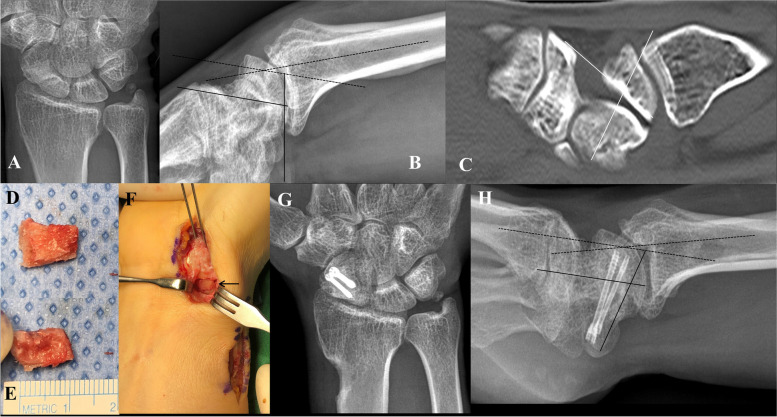
Corticocancellous bone graft from the anterolateral metaphysis of the distal radius (DR) and fixation of unstable scaphoid (Sc) nonunion. **A** and **B**) Posteroanterior and lateral preoperative radiographs of a 48-year-old man with unstable Sc nonunion (scapholunate angle [SLA, between 2 straight lines], 96°; radiolunate angle [RLA, between 2 dotted lines], 20°). **C**) Sagittal computed tomography scan showing humpback deformity of the Sc (intrascaphoid angle 89°). **D**, **E**) A wedge-shaped graft measuring 1 cm long and 0.6 cm wide obtained from the anterolateral metaphysis of the DR. **F**) The gap is filled with a wedge-shaped corticocancellous graft (arrow). **G**, **H**) Postoperative radiographs (SLA, 55°; RLA, 8°). Scaphoid union and deformity correction are confirmed by radiography. At 1 year postoperatively, the scaphoid has healed, and the donor site appears fully remodeled

Bone graft from the anterolateral metaphysis of the DR (Fig. 1).

All surgeries were performed with patients under regional anesthesia. The scaphoid was approached using a previously described palmar approach [15]. After the capsule was opened longitudinally, the site of nonunion was identified, and interposed fibrous tissue was excised using a burr until viable bleeding was observed. After preparing the nonunion site, the scaphoid shape and length were restored with 1.6-mm Kirschner (K)-wire joysticks. After scaphoid reduction, a guide wire and derotational K-wire were inserted to maintain the position of the scaphoid. Dimensions of the wedge graft were determined by measuring the gap in the scaphoid.

To harvest the graft, a separate anterolateral incision was made longitudinally from 1 cm proximal to the radial styloid. The deep fascia was released longitudinally to expose the lateral insertion of the pronator quadratus (PQ), which was detached from its lateral insertion and elevated subperiosteally. A small mini-Hohmann retractor was placed under the tendons of the first extensor compartment while protecting the radial artery and superficial branches of the radial nerve. After exposing the anterolateral border, a distal cut was made with an oscillating saw. To prevent radial styloid fracture, distal cut should be made parallel to the articular surface at least 2 cm proximal to the tip of the styloid process. A proximal cut was made to reproduce the shape and dimensions of the previously measured scaphoid defect. To prevent iatrogenic fracture of the styloid, the distance between the distal and proximal osteotomy sites needs to be longer than the depth of the osteotomy cut. After corticocancellous bone harvesting (Fig. 1D, E), the nonunion was fixed with 1 or 2 headless compression screws (Fig. 1F). 2.2-mm or 3.0-mm headless compression screw (Medartis, Basel, Switzerland) were used for scaphoid internal fixation. The donor site defect was covered by suturing the origin of the PQ to the BR tendon.

**Fig. 2:**
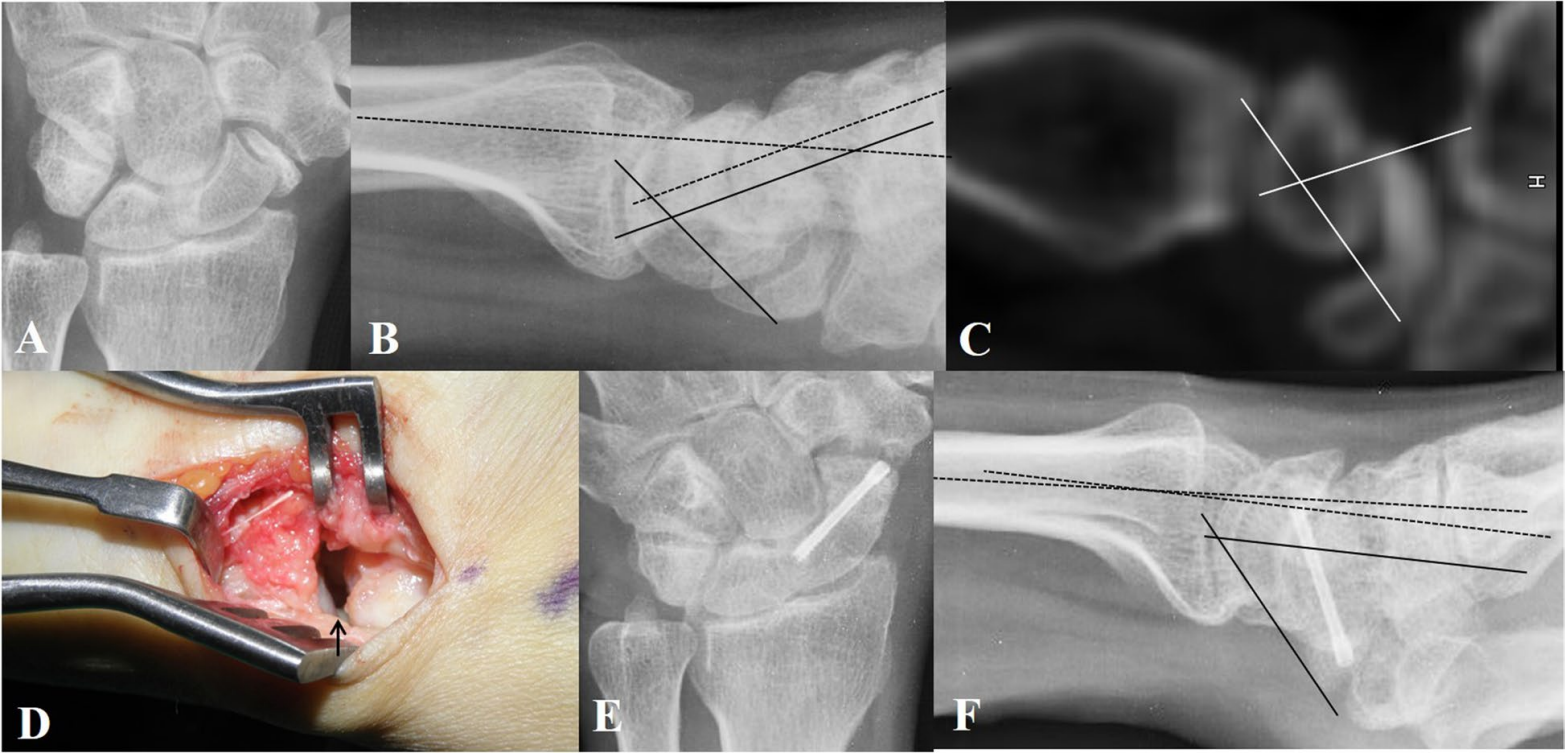
Corticocancellous bone grafting from the iliac crest and fixation of unstable scaphoid (Sc) nonunion. **A**, **B** and **C** Posteroanterior and lateral preoperative radiography and sagittal computed tomography of a 39-year-old man with unstable Sc nonunion (scapholunate angle [SLA, between 2 straight lines], 70°; radiolunate angle [RLA, between 2 dotted lines], 22°, Intrascaphoid angle [ISA, between 2 white lines], 71°). **D** The nonunion gap (arrowheads) is filled with corticocancellous bone from the iliac crest. **E, F** Postoperative radiographs (SLA, 50°; RLA, 3°)

Corticocancellous bone graft from the IC (Fig. 2).

The patient was placed under general anesthesia. The surgical approach, reduction technique, and screw fixation technique were the same as described above. To harvest the graft, an incision was made along the subcutaneous border of the IC at the point of contact with the periosteum and the origins of the gluteal and trunk muscles, and the incision was carried down to the bone. Using an osteotome, the structural graft to be removed was marked, and a power saw was used to cut the graft. The periosteum was sutured back, and the wound was closed in layers.

The same postoperative management protocol was adopted in the 2 groups. Patients were immobilized in a short-arm thumb spica cast for 6 to 8 weeks, and after cast removal, gentle exercises and light activities were permitted.

### Evaluation of outcomes

For radiologic evaluation, bony union and the lateral ISA, RLA, and SL were measured in each group. All patients underwent CT of the scaphoid and plain radiography of the wrist preoperatively. Plain radiographs were also obtained at 2-week intervals from 6 to 12 weeks postoperatively and then at 1-month intervals. Four radiographic views of the wrist, including true wrist images (posteroanterior, lateral, posteroanterior with ulnar deviation, and oblique with 45° pronation), were obtained at each follow-up visit. CT images were obtained using the technique described by Sanders [16]. When wrist radiographs suggested union, a longitudinal CT scan of the scaphoid was obtained to evaluate union and assess correction of the scaphoid humpback deformity. Bony union was assessed from the serial radiographs taken at each follow-up visit. Criteria for union included the absence of snuffbox tenderness and the presence of bridging trabeculae on the posteroanterior, scaphoid, lateral, and oblique wrist radiographs. CT was performed in all patients, and union was confirmed in each patient. Three authors (1 radiologist and 2 independent orthopedic surgeons) analyzed the following radiographic parameters of carpal alignment: ISA, SLA, and RLA. The scaphoid axis was defined as the tangent connecting the two palmar convexities of the bone. The lunate axis was defined as being perpendicular to the line joining the two distal horns of the lunate. The axis of the radius was obtained by tracing two centers of the medulla at 2 and 6 cm proximal to the radiocarpal joint and then connecting the centers. The SLA was defined as the angle between the scaphoid and lunate axes, RLA was defined as the angle between the longitudinal axes of the radius and lunate, radioscaphoid angle was defined as the angle between the longitudinal axes of the radius and scaphoid, lateral intrascaphoid angle was defined as the angle between lines drawn perpendicular to the proximal and distal articular surfaces, and articular surface was identified by subchondral sclerotic bone or the continuation of the articular curve on CT scans.

At follow-up, an independent examiner evaluated all patients clinically. All patients underwent measurements of motion of both wrists and grip strength. Range of motion (ROM) was measured with a handheld goniometer. Significant restriction of ROM was defined as a flexion/extension arc < 45 [17]. Grip strength was measured twice on both sides with a Jamar dynamometer (Sammons Preston, Bolingbrook, IL); results were averaged and expressed as percentages. At the time of the last follow-up, all patients completed a Disability of Arm, Shoulder, and Hand (DASH) questionnaire [18], and the Modified Mayo Wrist score (MMWS) was determined [19]. Operative time was reviewed and compared between the groups. It was measured in minutes from the beginning of preparing and draping the patient to when the final dressing was applied after closure. Each patient was also assessed for any donor site complications throughout the follow-up period.

### Statistical analysis

All continuous variables are expressed as a median with interquartile range in parenthesis after testing for normality using the Shapiro–Wilk test. All discrete variables are expressed as a frequency or ratio. When the 2 groups were compared, the Mann–Whitney test was used to analyze continuous variables, and the chi-square text (or Fisher exact test) was used to analyze discrete variables. Three authors participated in measuring the radiographic images, and the inter-observer correlation was good or excellent for each measure (Cronbach alpha, 0.893–0.931). The postoperative state was compared to the preoperative state using the Wilcoxon signed-rank test. *P* < 0.05 was considered statistically significant. MedCalc (version 11.6, MedCalc Software, Mariakerke, Belgium) and R (version 3.4.2, Comprehensive R Archive Network, GNU General Public License, Boston, MA) were used for all statistical analyses.

## Results

One hundred twelve patients underwent operative treatment for scaphoid nonunion in our institution during the study period. According to the inclusion criteria, 92 patients were screened (18 underwent only cancellous bone grafting and 2 underwent only K-wire fixation). Fifty-four patients were excluded (stable nonunions, *n* = 17; necrosis of the proximal fragment, *n* = 5; stage I or higher SNAC, *n* = 15; revision bone grafting surgery, *n* = 7; < 2 years of follow-up, *n* = 10). Of the 38 patients included in the study cohort, 15 underwent corticocancellous bone grafting of the anterolateral metaphysis of the DR (group DR), and 23 underwent bone grafting of the IC (group IC).

Patients’ characteristics are summarized in Table 1. No differences in demographic characteristics were found between the groups.

**Table 1:** Patients’ characteristics

Variable	Group DR (*n* = 15)	Group IC (*n* = 23)	*P*-value
Age (years)	28.0 (IQR: 18.0–35.8)	26.0 (IQR: 20.3–38.0)	.765
Sex (M/F)	15:0	23:0	.999
Interval of injury to surgery (years)	1.4 (1.1–2.9)	1.5 (IQR:1.0–2.8)	.495
Pre-treatment methods (none/splint or cast)	11:4	12:11	.335
Smoker (yes/no)	8/7	13/10	1.000
Number of screws used (1/2)	9/6	12/11	.888
Follow-up period, y	2.3 (IQR: 2.0–2.8)	2.4 (IQR: 2.0–3.2)	.949
Preoperative ISA, °	68.0 (IQR: 60.8–70.0)	65.0 (IQR: 56.3–72.8)	.822
Preoperative RLA, °	33.0 (IQR: 29.3–35.0)	30.0 (IQR: 23.3–33.8)	.072
Preoperative SLA, °	76.0 (IQR: 70.5–77.8)	72.0 (IQR: 70.0–76.5)	.208

Values are expressed as a median with interquartile range (IQR) in parenthesis for continuous variables and as a frequency or ratio for discrete variables

The chi-square test (or Fisher exact test) was used to analyze discrete variables, and the Mann–Whitney test was used to analyze continuous variables

Group DR, corticocancellous bone graft from the anterolateral metaphysis of the distal radius; group IC, corticocancellous bone graft from the iliac crest; *M* male, *F* female, *ISA* intrascaphoid angle, *RLA* radiolunate angle, *SLA* scapholunate angle

The operative time (minutes) was significantly shorter in group DR than in group IC (median, 98; quartiles, 80, 114 vs. median, 125; quartiles, 105, 150; *P* < 0.001).

**Fig. 3:**
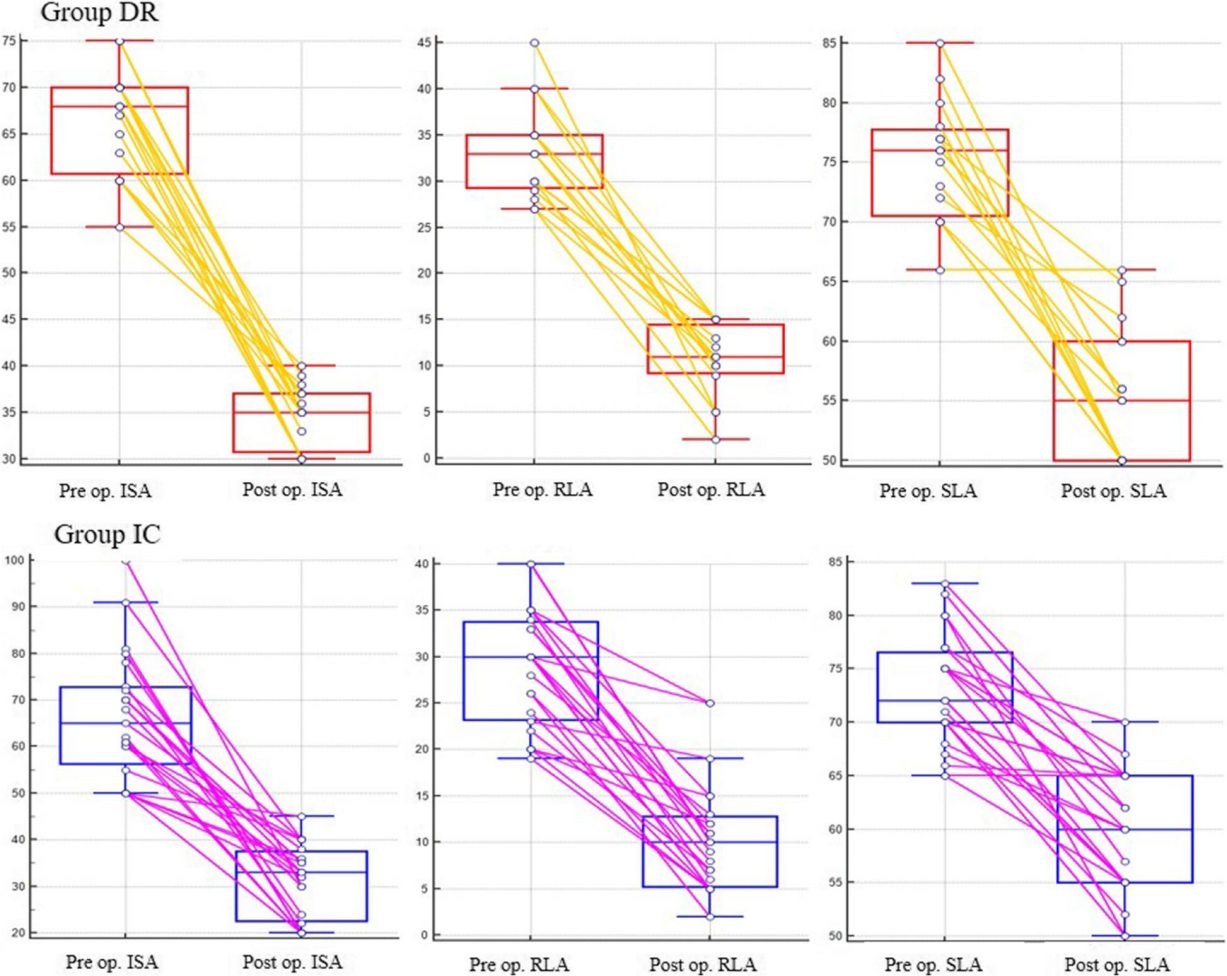
Restoration of carpal alignment according to preoperative and postoperative radiographic carpal alignment indices. Preop, preoperatively; Postop, postoperatively; ISA, intrascaphoid angle; RLA, radiolunate angle; SLA, scapholunate angle

All 15 patients in group DR and 22 of 23 (95.7%) patients in group IC achieved bone union postoperatively (*P* = 0.827). Mean time to union was not significantly different between the groups. All radiographic variables significantly improved postoperatively in both groups (*P* < 0.001) (Fig. 3).

**Fig. 4:**
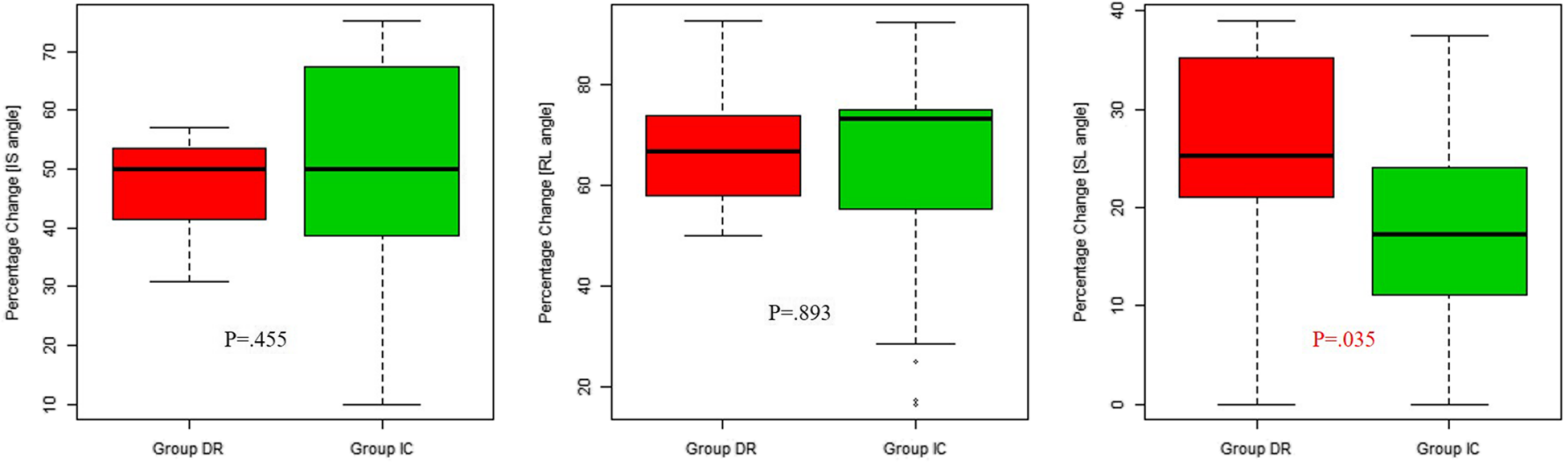
Comparisons of the percentage change of each radiographic variable between group DR and group IC. Percentage change of each radiographic variable was defined as the preoperative angle minus the postoperative angle divided by the preoperative angle. Statistical analysis was performed using the Mann–Whitney test. IS, intrascaphoid; RL, radiolunate; SL, scapholunate

To assess the corrective capacity for deformity between groups, the percentage change of each radiographic variable was defined as the preoperative angle minus the postoperative angle divided by the preoperative angle. SLA showed superior deformity correction capability in group DR compared with that in group IC (25.3% vs. 17.3%, *P* = 0.035), but the ISA and RLA were not significantly different between the groups (Fig. 4).

The number of patients with significant residual deformity postoperatively (%) was not significantly different between the groups (ISA > 35°, 40% vs. 30.4%, *P* = 0.728; RLA > 15°, 0% vs. 13%, *P* = 0.264; SLA > 60, 20% vs. 47.8%, *P* = 0.100).

No significant differences were found between the groups in the incidence of significant restriction of ROM, grip strength, MWS, and DASH score postoperatively (Table 2).

**Table 2:** Comparisons of the clinical outcomes between group DR and group IC

	Group DR	Group IC	*P*-value
% Significant restriction of ROM	0/15 (0%)	2/23 (8.7%)	.509
Modified Mayo wrist score	95 (IQR: 90–100)	90 (IQR: 85–100)	.187
Grip strength (%)	85.4 (IQR: 81–100)	86.4 (IQR: 83–100)	.283
DASH score	5.2 (IQR: 3.0–6.0)	5.5 (IQR: 3.0–7.5)	.917

Values are expressed as a median with an interquartile range (IQR) in parenthesis for continuous variables and as a frequency or ratio for discrete variables

*ROM* range of motion, *DASH* Disability of Arm, Shoulder, and Hand; group DR, corticocancellous bone graft from the anterolateral metaphysis of the distal radius; group IC, corticocancellous bone graft from the iliac crest

No significant differences in donor site complications were found between the groups, but the overall complication rate at the donor site was higher in group IC than in group DR (26% vs. 0%, *P* = 0.063). In group IC, 2 patients had superficial hematoma, and 3 had persistent graft site pain. In group DR, 1 patient had superficial radial nerve irritation symptom, but it resolved at 2 months postoperatively (Table 3).

**Table 3:** Comparisons of donor site complications between group DR and group IC

	Group DR	Group IC	*P*-value
Persistent pain	0	4 (17.3%)	.264
Hematoma	0	2 (8.6%)	.509
Infection	0	0	.999
Nerve irritation	0	0^a^	.999
Graft site fracture	0	0	.999
Overall	0 (0%)	6 (26%)	.063

^a^One patient had transient nerve irritation in group DR, but this was not counted because the complication resolved spontaneously within 6 weeks postoperatively

Group DR, corticocancellous bone graft from the anterolateral metaphysis of the distal radius; group IC, corticocancellous bone graft from the iliac crest

## Discussion

This study demonstrates that corticocancellous bone graft from the anterolateral metaphysis of the DR not only showed similar union rate, restorations of scaphoid deformity and wrist function but also time efficient compared to corticocancellous bone graft from the IC when scaphoid nonunion was treated by headless compression screw fixation.

The goal of treatment for scaphoid nonunion are to achieve union, restore carpal alignment and prevent progressive arthritis. Several open and arthroscopic approaches for nonunion have been reported. Recently, arthroscopic approach of scaphoid nonunion which is known for minimal invasive technique reports similar bone union rates and clinical results compared to open approach [20, 21]. However, questions about the possibility of restoring carpal alignment remain [21]. Several bone grafting techniques have been described using corticocancellous or cancellous-only grafts [1, 22–25]. Many studies suggested that cancellous bone graft provide early bone union and similar anatomic restoration compared to corticocancellous bone graft [26, 27]. However, corticocancellous bone grafts are associated with consistent deformity correction and therefore it is recommended in patients with high degree of scaphoid humpback deformity [28]. To enhance the stability of fracture fixation, a compression screw in 1984 by Herbert and Fisher for the treatment of scaphoid fractures [29]. Since then, newer generations of screws, memory staple [30], and plate [31] have been developed. Additionally, biological factors such as platelet-rich plasma also being applied to improve bone healing [30, 32].

Christodoulou et al. showed no statistical difference in the time to union or union rate between IC and DR grafts [13]. Our study also showed that the union rate and time to union were not significantly different between the grafts. Jarrett et al. showed comparable biomechanical strength between the IC and DR bone grafts [33]. According to some authors, taking the graft from the dorsal aspect of the DR at the Lister tubercle can be as effective as taking it from the IC [11, 12, 24]. However, the dorsal cortex at the Lister tubercle has a thin cortex, and there is a void of the medullary trabecular bone beneath the cortical bone, leaving only a small portion of cancellous bone. Conversely, the anterolateral metaphysis of the DR involves a thicker cortical bone than the dorsal cortex and cancellous bone is suitably dense [34]. In our practice, all patients’ bone defect sizes after excision of the nonunion site were within 1.5 cm, and a sufficient amount of bone was available in the DR.

Our study showed that corticocancellous bone graft from the anterolateral metaphysis of the DR and IC could restore carpal alignment. Deformity can be corrected by corticocancellous bone graft from the IC [23, 35], but radiologic outcomes confirming the ability of deformity correction in DR bone graft are difficult to find in the literature. According to Tambe et al., a similar postoperative SLA was seen with both bone graft techniques [11]. However, there was no preoperative measurement; thus, the degree of correction was not confirmed. Moreover, the deformity correction in some cases containing a proximal pole nonunion could not be accurately evaluated. A randomized controlled trial by Garg et al. has demonstrated no significant difference was observed in postoperative mean SLA (50° in DR and 54° in IC) between the corticocancellous graft from DR and IC at 3-year follow-up [12]. According to our results, regarding the capability of deformity correction evaluated with the SLA, DR bone graft was better in restoring normal scaphoid alignment, but there was no difference between the RLA and ISA. To investigate the deformity and reduction of the scaphoid itself, the SLA should be the most effective criterion [1, 36]. However, previous studies [37, 38] showed that among carpal alignment indices, the RLA is the most reliable and valid carpal alignment index for evaluating the deformities of scaphoid nonunions, and it correlated with clinical outcome after reconstruction. Thus, we could not verify the superiority of the correction capability of 2 bone graft sources with only SLA.

Previous studies have reported minor complications after IC bone grafting in 7.1% to 39% of patients and major complications in 1.8% to 10% of patients [7, 8, 39, 40]. In our study, there were no major complications in group IC, but there were 2 cases of superficial hematoma and 3 cases of persistent graft site pain. A possible complication in graft harvesting from the anterolateral DR is graft site fracture. In order to prevent this complication, graft was obtained 2.5 cm proximal from the radial styloid, and the distal cut was made parallel to the articular surface of the radius [10]. Additionally, immobilization for 6 to 8 weeks was prescribed to promote bone ingrowth in the defect. In all cases, complete healing of the donor site defect was observed by radiography at 6 months to 1 year later.

Herein, bone grafting of the DR was associated with significantly shorter operative times than bone grafting of the IC. We inferred that this finding is attributed to the fact that the DR bone graft procedure has donor and recipient sites in the same surgical field and preparation of the operation is relatively simple compared with the IC bone graft procedure. The DR bone graft technique can reduce the time to graft harvesting, allowing the surgeon to concentrate on the main procedure without worrying about the tourniquet time. Moreover, regional anesthesia is possible, which can be beneficial in selected patients [41, 42], increase operating room efficacy [43, 44] and enable outpatient treatment.

Our study has several limitations. First, it included a small number of patients and had low statistical power, thereby increasing the chance of a type II error. Second, this was a retrospective study, not a randomized, controlled trial. Thus, there may be selection bias of patient enrollment for each surgical technique. A prospective, randomized study that compares the two bone graft techniques should be performed. Lastly, a comparison of the effects of a brachial plexus block and general anesthesia on postoperative subjective pain and duration of hospitalization was not performed; hence, future studies should address this topic.

In conclusion, corticocancellous bone graft from the anterolateral metaphysis of the DR can be effectively used to treat scaphoid unstable nonunions with humpback deformity, with a reliable radiologic outcome and relatively shorter operative time than IC bone graft.

## Data Availability

The datasets used and/or analysed during the current study available from the corresponding author on reasonable request.

## References

[1] Fernandez DL (1984). A technique for anterior wedge-shaped grafts for scaphoid nonunions with carpal instability. J Hand Surg Am.

[2] Fisk GR (1970). Carpal instability and the fractured scaphoid. Ann R Coll Surg Engl.

[3] Sayegh ET, Strauch RJ (2014). Graft choice in the management of unstable scaphoid nonunion: a systematic review. J Hand Surg Am.

[4] Rajagopalan BM, Squire DS, Samuels LO (1999). Results of Herbert-screw fixation with bone-grafting for the treatment of nonunion of the scaphoid. J Bone Joint Surg Am.

[5] Hull WJ, House JH, Gustillo RB, Kleven L, Thompson W (1976). The surgical approach and source of bone graft for symptomatic nonunion of the scaphoid. Clin Orthop Relat Res.

[6] Filan SL, Herbert TJ (1996). Herbert screw fixation of scaphoid fractures. J Bone Joint Surg Br.

[7] Arrington ED, Smith WJ, Chambers HG, Bucknell AL, Davino NA (1996). Complications of iliac crest bone graft harvesting. Clin Orthop Relat Res.

[8] Goulet JA, Senunas LE, DeSilva GL, Greenfield ML. Autogenous iliac crest bone graft. Complications and functional assessment. Clin Orthop Relat Res. 1997(339):76–81. 10.1097/00003086-199706000-00011 9186204

[9] Smith SE, DeLee JC, Ramamurthy S. Ilioinguinal neuralgia following iliac bone-grafting. Report of two cases and review of the literature. J Bone Joint Surg Am. 1984;66(8):1306–8. 6386820

[10] Aguilella L, Garcia-Elias M (2012). The anterolateral corner of the radial metaphysis as a source of bone graft for the treatment of scaphoid nonunion. J Hand Surg Am.

[11] Tambe AD, Cutler L, Murali SR, Trail IA, Stanley JK (2006). In scaphoid non-union, does the source of graft affect outcome? Iliac crest versus distal end of radius bone graft. J Hand Surg Br.

[12] Goyal T, Sankineani SR, Tripathy SK (2013). Local distal radius bone graft versus iliac crest bone graft for scaphoid nonunion: a comparative study. Musculoskelet Surg.

[13] Christodoulou LS, Kitsis CK, Chamberlain ST (2001). Internal fixation of scaphoid non-union: a comparative study of three methods. Injury.

[14] Schmidle G, Ebner HL, Klima G, Pfaller K, Fritz J, Hoermann R, Gabl M (2018). Time-dependent changes in bone healing capacity of scaphoid fractures and non-unions. J Anat.

[15] Kawamura K, Chung KC (2008). Treatment of scaphoid fractures and nonunions. J Hand Surg Am.

[16] Sanders WE (1988). Evaluation of the humpback scaphoid by computed tomography in the longitudinal axial plane of the scaphoid. J Hand Surg Am.

[17] Brumfield RH, Champoux JA (1984). A biomechanical study of normal functional wrist motion. Clin Orthop Relat Res.

[18] Hudak PL, Amadio PC, Bombardier C. Development of an upper extremity outcome measure: the DASH (disabilities of the arm, shoulder and hand) [corrected]. The Upper Extremity Collaborative Group (UECG). Am J Ind Med. 1996;29(6):602–8. 10.1002/(sici)1097-0274(199606)29:6%3c602::Aid-ajim4%3e3.0.Co;2-l. 10.1002/(SICI)1097-0274(199606)29:6<602::AID-AJIM4>3.0.CO;2-L 8773720

[19] Cooney WP, Bussey R, Dobyns JH, Linscheid RL. Difficult wrist fractures. Perilunate fracture-dislocations of the wrist. Clin Orthop Relat Res. 1987(214):136–47. 3791735

[20] Slade JF, Dodds SD (2006). Minimally invasive management of scaphoid nonunions. Clin Orthop Relat Res.

[21] Oh WT, Kang HJ, Chun YM, Koh IH, Lee YJ, Choi YR (2018). Retrospective comparative outcomes analysis of arthroscopic versus open bone graft and fixation for unstable scaphoid nonunions. Arthroscopy.

[22] Bindra R, Bednar M, Light T (2008). Volar wedge grafting for scaphoid nonunion with collapse. J Hand Surg Am.

[23] Cooney WP, Linscheid RL, Dobyns JH, Wood MB (1988). Scaphoid nonunion: role of anterior interpositional bone grafts. J Hand Surg Am.

[24] Cohen MS, Jupiter JB, Fallahi K, Shukla SK (2013). Scaphoid waist nonunion with humpback deformity treated without structural bone graft. J Hand Surg Am.

[25] De Vitis R, Passiatore M, Perna A, Tulli A, Pagliei A, Taccardo G (2020). Modified Matti-Russe technique using a “butterfly bone graft” for treatment of scaphoid non-union. J Orthop.

[26] Kim JK, Yoon JO, Baek H (2018). Corticocancellous bone graft vs cancellous bone graft for the management of unstable scaphoid nonunion. Orthop Traumatol Surg Res.

[27] Hegazy G, Massoud AH, Seddik M, Abd-Elghany T, Abdelaal M, Saqr Y, Abdelaziz M, Zayed E, Hassan M (2021). Structural versus nonstructural bone grafting for the treatment of unstable scaphoid waist nonunion without avascular necrosis: a randomized clinical trial. J Hand Surg Am.

[28] Van Nest D, Ilyas AM (2022). Scaphoid nonunion: a review of surgical strategies. Orthopedics.

[29] Herbert TJ, Fisher WE (1984). Management of the fractured scaphoid using a new bone screw. J Bone Joint Surg Br.

[30] De Vitis R, Passiatore M, Perna A, Fioravanti Cinci G, Taccardo G (2019). Comparison of shape memory staple and gelled platelet-rich plasma versus shape memory staple alone for the treatment of waist scaphoid nonunion: a single-center experience. Joints.

[31] Leti Acciaro A, Lana D, Fagetti A, Cherubino M, Adani R (2022). Plate fixation in challenging traumatic carpal scaphoid lesions. Musculoskelet Surg.

[32] Zhong Z, Wei M, Jiang Z, Chen J, He Y, Lin K (2023). Comparative effectiveness of three treatment options for slade and dodds grade III-IV scaphoid nonunion: a retrospective study. BMC Musculoskelet Disord.

[33] Jarrett P, Kinzel V, Stoffel K (2007). A biomechanical comparison of scaphoid fixation with bone grafting using iliac bone or distal radius bone. J Hand Surg Am.

[34] Bain GI, MacLean SBM, McNaughton T, Williams R (2017). Microstructure of the distal radius and its relevance to distal radius fractures. J Wrist Surg.

[35] Nakamura P, Imaeda T, Miura T (1991). Scaphoid malunion. J Bone Joint Surg Br.

[36] Tsuyuguchi Y, Murase T, Hidaka N, Ohno H, Kawai H. Anterior wedge-shaped bone graft for old scaphoid fractures or non-unions. An analysis of relevant carpal alignment. J Hand Surg Br. 1995;20(2):194–200. 10.1016/s0266-7681(05)80049-x 7797969

[37] Megerle K, Harenberg PS, Germann G, Hellmich S (2012). Scaphoid morphology and clinical outcomes in scaphoid reconstructions. Injury.

[38] Roh YH, Noh JH, Lee BK, Baek JR, Oh JH, Gong HS, Baek GH (2014). Reliability and validity of carpal alignment measurements in evaluating deformities of scaphoid fractures. Arch Orthop Trauma Surg.

[39] Morgan SJ, Jeray KJ, Saliman LH, Miller HJ, Williams AE, Tanner SL, Smith WR, Broderick JS. Continuous infusion of local anesthetic at iliac crest bone-graft sites for postoperative pain relief. A randomized, double-blind study. J Bone Joint Surg Am. 2006;88(12):2606–12. 10.2106/jbjs.E.00984. 10.2106/JBJS.E.00984 17142410

[40] Myeroff C, Archdeacon M (2011). Autogenous bone graft: donor sites and techniques. J Bone Joint Surg Am.

[41] Hustedt JW, Chung A, Bohl DD, Olmschied N, Edwards SG (2017). Comparison of postoperative complications associated with anesthetic choice for surgery of the hand. J Hand Surg Am.

[42] Hadzic A, Arliss J, Kerimoglu B, Karaca PE, Yufa M, Claudio RE, Vloka JD, Rosenquist R, Santos AC, Thys DM (2004). A comparison of infraclavicular nerve block versus general anesthesia for hand and wrist day-case surgeries. Anesthesiology.

[43] Mariano ER, Chu LF, Peinado CR, Mazzei WJ (2009). Anesthesia-controlled time and turnover time for ambulatory upper extremity surgery performed with regional versus general anesthesia. J Clin Anesth.

[44] Williams BA, Kentor ML, Williams JP, Figallo CM, Sigl JC, Anders JW, Bear TC, Tullock WC, Bennett CH, Harner CD (2000). Process analysis in outpatient knee surgery: effects of regional and general anesthesia on anesthesia-controlled time. Anesthesiology.

